# Clinical and Serological Evaluation of LINDA Virus Infections in Post-Weaning Piglets

**DOI:** 10.3390/v11110975

**Published:** 2019-10-23

**Authors:** Alexandra Kiesler, Kerstin Seitz, Lukas Schwarz, Katharina Buczolich, Helga Petznek, Elena Sassu, Sophie Dürlinger, Sandra Högler, Andrea Klang, Christiane Riedel, Hann-Wei Chen, Marlene Mötz, Peter Kirkland, Herbert Weissenböck, Andrea Ladinig, Till Rümenapf, Benjamin Lamp

**Affiliations:** 1 Institute of Virology, Department for Pathobiology, University of Veterinary Medicine, Veterinaerplatz 1, 1210 Vienna, Austria; Alexandra.Kiesler@vetmeduni.ac.at (A.K.); Kerstin.Seitz@vetmeduni.ac.at (K.S.); katharina.buczolich@gmx.net (K.B.); Helga.Petznek@vetmeduni.ac.at (H.P.); Christiane.Riedel@vetmeduni.ac.at (C.R.); Hann-Wei.Chen@vetmeduni.ac.at (H.-W.C.); Marlene.Moetz@vetmeduni.ac.at (M.M.); Till.Ruemenapf@vetmeduni.ac.at (T.R.); 2 Department for Farm Animals and Veterinary Public Health, University Clinic for Swine, University of Veterinary Medicine, Veterinaerplatz 1, 1210 Vienna, Austria; Lukas.Schwarz@vetmeduni.ac.at (L.S.); Elena.Sassu@vetmeduni.ac.at (E.S.); Sophie.Duerlinger@vetmeduni.ac.at (S.D.); Andrea.Ladinig@vetmeduni.ac.at (A.L.); 3 Institute of Pathology and Forensic Veterinary Medicine, Department of Pathobiology, University of Veterinary Medicine, Veterinaerplatz 1, 1210 Vienna, Austria; Sandra.Hoegler@vetmeduni.ac.at (S.H.); Andrea.Klang@vetmeduni.ac.at (A.K.); Herbert.Weissenboeck@vetmeduni.ac.at (H.W.); 4 Virology Laboratory, Elizabeth Macarthur Agriculture Institute, Woodbridge Rd, Menangle, New South Wales 2568, Australia; Peter.Kirkland@dpi.nsw.gov.au

**Keywords:** Linda virus, serological profile, virus neutralization assay, virus pathogenicity, humoral immune response

## Abstract

The novel pestivirus species known as lateral-shaking inducing neuro-degenerative agent (LINDA) virus emerged in 2015 in a piglet-producing farm in Austria. Affected piglets showed strong congenital tremor as a result of severe lesions in the central nervous system. Here, we report the results of a controlled animal infection experiment. Post-weaning piglets were infected with LINDA to determine the susceptibility of pigs, the clinical consequences of infection and the humoral immune response against LINDA. No clinically overt disease signs were observed in the piglets. Viremia was hardly detectable, but LINDA was present in the spleen and several lymphatic organs until the end of the experiment on day 28 post-infection. Oronasal virus shedding together with the infection of one sentinel animal provided additional evidence for the successful replication and spread of LINDA in the piglets. Starting on day 14 post-infection, all infected animals showed a strong humoral immune response with high titers of neutralizing antibodies against LINDA. No cross-neutralizing activity of these sera with other pestiviral species was observed. According to these data, following postnatal infection, LINDA is a rather benign virus that can be controlled by the pig’s immune system. However, further studies are needed to investigate the effects of LINDA on the fetus after intrauterine infection.

## 1. Introduction

The genus *Pestivirus* within the family Flaviviridae currently comprises 11 different species—recently termed *Pestivirus A–K* [1]. In addition to the long known classical swine fever virus (CSFV, *Pestivirus C*), a number of other pestivirus species have been identified in the porcine host in recent years, such as border disease virus (BDV, *Pestivirus D*), bovine viral diarrhea virus (BVDV-1, *Pestivirus A*), Bungowannah virus (BUNGO, *Pestivirus F*), and atypical porcine pestivirus (APPV, *Pestivirus K*) [1,2,3,4,5]. In 2015, we detected a yet unknown pestivirus species in a piglet-producing farm in Austria, which was termed lateral-shaking inducing neuro-degenerative agent (LINDA) virus [6]. Since the nucleotide sequence of LINDA shows a significant divergence of over 30% compared to the accepted pestivirus species, we proposed it as the new species *Pestivirus L* [1,6].

Pestiviruses are small enveloped viruses with a positive-sense, single-stranded, non-segmented RNA genome with a length of about 12 to 13 kilobases (kb) [7]. The genome consists of one large open reading frame (ORF), flanked by 5′- and 3′-non-coding regions [7]. This single ORF encodes a hypothetical polyprotein, that is co- and post-translationally processed into non-structural and structural proteins by viral and cellular proteases [8]. The three structural glycoproteins, termed E^rns^, E1 and E2, and the nucleocapsid protein named Core are generated by cellular proteases [9,10]. The generation of the non-structural proteins N^pro^, p7, NS2, NS3, NS4A, NS4B, NS5A and NS5B is very complex. Multiple processing steps mediated by autoproteases (N^pro^ and NS2) and the major NS3/4A protease yield partially processed precursors, mature proteins and enzymatically active protein fragments [8,11,12,13]. The presence of the autoprotease N^pro^ and the envelope glycoprotein E^rns^ are recognized as characteristic of the genus *Pestivirus* [1,7]. Since the corresponding proteins have been found in the genome of LINDA, it can undoubtedly be classified in the genus *Pestivirus* [6].

CSFV is listed by the World Organization for Animal Health (OIE) as an economically important pig pathogen [14]. The clinical signs of classical swine fever (CSF) vary significantly depending on the virulence of the virus strain as well as the age and susceptibility of the infected pigs. CSF is usually characterized by fever, skin lesions, convulsions and, especially in young animals, death within a few days [15]. BUNGO emerged on a pig farm in Australia in 2003, causing an increased rate of stillbirths, mummification and sudden deaths of piglets [2,16]. Experimental studies were conducted to investigate the pathogenicity of BUNGO in weaner pigs and porcine fetuses under laboratory conditions. Despite the low pathogenicity of the virus in weaned piglets, a long-lasting viremia, efficient virus shedding and rapid seroconversion were detected [17]. In contrast, a multifocal non-suppurative myocarditis with myonecrosis was observed following direct fetal exposure to BUNGO mimicking intrauterine infection [18]. APPVs were discovered in the United States in 2015 by next-generation sequencing [4], and subsequently detected in many countries around the world [19,20,21,22,23]. A close correlation between intrauterine APPV infections and the occurrence of congenital tremor (CT) type A-II in newborn piglets was reported [24]. The simultaneous detection of nucleic acids of APPV and hypomyelination in the central nervous system of these piglets implied a causative role of APPV for the appearance of the so-called shaking piglet syndrome [20]. This causal relationship is further supported by the birth of shaking piglets after inoculation of pregnant sows with APPV-containing material [24].

LINDA was discovered during the investigation of an outbreak of CT in a piglet-producing farm. We identified the agent, isolated the virus, sequenced its genome and established a RT-PCR assay as well as serological reagents for its detection [6]. Since then, LINDA has not been found in any other farm in Austria or elsewhere in the world [25]. To gain a deeper insight into the biology of this virus, we infected weaned piglets with LINDA under controlled experimental conditions. The aim of this small-scale animal experiment was the determination of susceptibility, pathogenicity and virulence of LINDA in the immunocompetent porcine host. Sera from the experimentally infected piglets were further used to characterize the humoral immune response against LINDA and to study the induction of cross-neutralizing antibodies against other pestiviruses.

## 2. Materials and Methods 

### 2.1. Cells and Viruses

SK-6 cells [26] and MDBK cells (ATCC^®^ CCL-22™) [27] were grown in Dulbecco’s modified Eagle’s medium (DMEM) supplemented with 10% fetal bovine serum (FCS, Bio and Sell GmbH, Feucht, Germany; negatively tested for the presence of pestiviruses), 100 U/mL penicillin and 100 µg/mL streptomycin. All cells were maintained at 37 °C and 5% CO_2_. Cell culture-derived LINDA was used for the experiments. After initial cell culture isolation from a clinical case (passage 1, P1), a primary LINDA stock was generated containing a 50% tissue culture infectious dose (TCID_50_) of 1.1 × 10^7^ (P2, GenBank^®^ KY436034) [6]. The virus was titrated in an endpoint dilution assay and supernatant from a single focus was harvested (P3) to ensure freedom from other pathogens. A master stock (P4) was prepared and characterized by RT-PCR and subsequent Sanger sequencing. In direct comparison with the consensus sequence of the original isolate no mutations were detected. All LINDA infection doses used for animal inoculations were recovered from the master stock and thus represent cell culture passage 5 of LINDA. BVDV-1a strain NADL and BVDV-1b strain NCP7 were obtained from E. Dubovi (Cornell University College of Veterinary Medicine, Ithaca, NY) [28]. CSFV 2.3 Alfort-Tübingen [29], BDV-1 X818 [30], BVDV-2 strain 890 [31], and BVDV-3 (unpublished strain isolated from FCS, South American origin) were obtained from the virus collection of the Institute of Virology in Giessen (Justus-Liebig-University, Giessen, Germany). Pestivirus strain Giraffe-1 [32] was a gift from D. J. Paton, Animal Health and Veterinary Laboratory Agency (AHVLA, Weybridge, United Kingdom). BUNGO was obtained from stocks of the Elizabeth Macarthur Agricultural Institute (Department of Primary Industries, Menangle, New South Wales, Australia) [2].

### 2.2. Virus Infection and Titration

Infections of MDBK and SK-6 cells with various strains of the different pestivirus species were performed with the indicated multiplicity of infection (MOI). Virus stocks for the experiments were generated using 10-cm cell culture dishes infected with a MOI of 0.1. At 72 h post-infection, the cell culture supernatant was harvested, filtered through a 0.45 µm cellulose filter (Sartorius, Göttingen, Germany), aliquoted and stored at −80 °C until use. The TCID_50_ of viral supernatants was determined in three replicates by an end-point dilution assay (EPDA). The virus titer was calculated using the Spearman-Kaerber algorithm [33]. Re-isolation of LINDA was performed using SK-6 cells and fresh sample material.

### 2.3. RT-PCR Detection

RNA was extracted from serum and tissue samples, saliva, feces, cultured cells or virus cell culture supernatant using the QIAamp Viral RNA Mini Kit and the RNeasy Mini Kit (Qiagen, Hilden, Germany) according to the manufacturer’s instructions. RNA was eluted in 60 µL RNase free distilled water and directly used for RT-PCR or stored at –80 °C for subsequent analysis. RT-PCR was carried out using the OneTaq One-Step RT-PCR Kit (NEB, Ipswich, USA) or the One Step RT-PCR Kit (Qiagen) using the oligonucleotides PPF 5′-GTKATHCAATACCCTGARGC-3′ and PPR 5′-GGRTTCCAGGARTACATCA-3′ [6]. PCR amplicons were subjected to gel electrophoresis and purified with the peqGOLD Gel Extraction Kit (Peqlab, Erlangen, Germany), if needed.

### 2.4. Calibration-Curve-Estimated Copy Number of LINDA Genome Equivalents

For the quantification of virus excretion, viremia and virus loads of organ homogenates, qRT-PCRs were performed on an ABI 7500 cycler (Applied Biosystems, Foster City, USA) using the LINDA and BUNGO specific primers LVqRTfor196 (5′-CACTGGWAAGGATCACCCACT-3′) and LVqRTrev351 (5′-AATYACAACGGATAWTMTTTATACTGG-3′) and the FAM/TAMRA labeled probe, LVqRTprobe322 (5′-Fam-ATAGGATGCCGGCGGATGCCCTGT-TamRa -3′). For the generation of a calibration curve, a T-vector plasmid fragment harboring the cDNA target sequence was produced using EcoRI digest, gel-purified and spectrophotometrically quantified. The copy number of recombinant plasmid fragment was calculated following the formula: N (molecules per µL) = (C (DNA concentration in µg/µL) / K (fragment size in bp)) × 185.5 × 10^13^. The factor results from the weight of the genome-equivalent ssDNA molecules (330 daltons per base), the volume projection factors and the Avogadro constant (6.02 × 10^23^ mol^–1^). In order to obtain a standard curve, a ten-fold dilution series of the DNA control was included in the qRT-PCR setup [34]. Cycling conditions were 50 °C for 10 min, 95 °C for 1 min and 40 cycles of 95 °C for 10 sec, 60 °C for 1 min (amplification and fluorescence detection step). Semi-quantitative copy number estimation was calculated by 7500 System SDS Software (Applied Biosystems) based on the calibration curve. The amplification of the dsDNA standard does not include reverse transcription, which is why minor deviations may occur between RNA samples and dsDNA standard amplification depending on the efficiency of the cDNA synthesis. The qRT-PCR assay presented here is linear up to 10 copies of the dsDNA template per reaction (Ct value of 38). Since 10 copies per reaction were included as the lowest template amount in the standard series, this value represents the limit of quantification and our lower assay cutoff. The genome equivalents of 1.0 µL of purified RNA were converted to copies per 1 mL liquid sample, 1 g tissue sample or copies per swab using appropriate projection factors. The samples from the animal experiment were measured once due to high numbers and limited financial resources. However, the qRT-PCR data were validated by virus isolation experiments carried out in parallel with all samples obtained during the experiment.

### 2.5. Virus Neutralization Assay

All virus neutralization assays (VNA) were prepared in triplicate in 96-well microtiter plates. Viruses used in the VNA were diluted in DMEM without FCS from stock solutions generating a final titer of 100 to 300 TCID_50_ per 0.1 mL. Initial two-fold dilution series of the serum samples were prepared with DMEM without FCS generating a final serum dilution of 1:256 in the last wells. Highly reactive sera were further diluted in five-fold series to a final serum dilution of 1:10^5.6^ in the last wells. 100 µL of each serum dilution were mixed with 100 μL of the respective virus solution containing between 100 to 300 TCID_50_. After the virus was added, the VNA was incubated for 2 h at 37 °C in 5% CO_2_. One hundred microliters of this virus/serum mixtures were added to 96-well flat bottom plates containing confluent cell monolayers and incubated for 48 h at 37 °C in 5% CO_2_. Viral infections were detected by indirect immunofluorescence using murine monoclonal antibodies (MAbs) as indicated below. Back titration of each virus solution was performed in parallel. Defined positive and negative sera against the respective virus or groups were used as controls. The titers obtained from the VNAs were calculated using the Spearman-Kaerber algorithm and reported as the reciprocal of the serum dilution that inhibited infection of 50% of the cells (neutralization dose 50%, ND_50_).

### 2.6. Indirect Immunofluorescence Assay and Antibodies

The immunofluorescence assays were performed as previously described [8]. Briefly, the cells were fixed with 4% paraformaldehyde for 20 min at 4 °C, permeabilized with 1% (vol/vol) Triton-X 100 (Merck, Darmstadt, Germany) in PBS and stained with the mouse MAb 6A5 [35] and A18 [36]. The monoclonal antibody 6A5 was used to detect the E2 molecule of BVDV-1, BVDV-2, BVDV-3, BDV, BUNGO, giraffe pestivirus and LINDA infections. CSFV E2 was detected by MAb A18. Goat anti-mouse IgG conjugated with Cy3 (Dianova, Hamburg, Germany) was used as a secondary antibody. A porcine BUNGO antiserum (748-09.10-1) collected from a naturally infected sow, which had produced an abnormal litter, was kindly provided by the Elizabeth Macarthur Agricultural Institute.

### 2.7. Animal Experiment

The animal experiment was approved by the ethics committee of the University of Veterinary Medicine, Vienna and the Federal Ministry of Science, Research and Economy according to the §§ 26ff. of the Austrian Animal Experiments Act from 2012 (Permission code: BMWF-68.205/0130-WF/V/3b/2017, permission date: 07.07.2017). Post-weaning crossbreed piglets (*Sus scrofa domestica*) were used for the study of LINDA pathogenesis and virulence in immunocompetent hosts. The pigs came from the breeding farm Medau of the University of Veterinary Medicine, Vienna. The entire herd of the farm was negative tested for swine pathogenic influenza A viruses (IAV; H3N2, H1N1, H1N2 and H1N1pan) and porcine reproductive and respiratory syndrome viruses (PRRSV). The mother sows of the experimental animals were vaccinated against parvovirus and erysipelas during lactation according to the manufacturer’s instructions (Parvoruvac; Merial GmbH, Hallbergmoos, Germany). The piglets themselves were protected on day 21 with a combined vaccination against *Mycoplasma hyopneumoniae* and porcine circovirus-2 (Circoflex and Mycoflex; both from Boehringer Ingelheim Vetmedica GmbH, Ingelheim, Germany). No further special diagnostic tests were carried out as there were neither pathological nor clinical signs of disease in these piglets.

A week prior to the beginning of the trial (study days –7 to –1) 21 weaned piglets in the 13th week of life were housed in a biological safety unit (BSL-2) for adaptation. At the beginning of the animal experiment, six piglets were housed separately in order to later serve as sentinel animals. The remaining piglets were divided into three groups of five animals each and housed in separate units. One group was not infected and served as a negative control, one group was inoculated intramuscularly (i.m.) with 1 × 10^7^ TCID_50_ LINDA and the last group was infected intranasally (i.n.) with 1 × 10^7^ TCID_50_ LINDA (study day 0). One day after infection (study day 1), three sentinel animals were added to each of the infection-groups. A daily clinical score was determined for each individual animal. The general condition, behavior, body temperature, feed intake and weight gain of all animals were assessed and measured after the infection over a period of 28 days. Particular attention was paid to the occurrence of signs of disease. Each animal was assigned a daily clinical score, which included individual values for behavior, feed intake, dyspnea, ocular and nasal discharge, coughing and diarrhea in the range from 0 (physiological) to 3 (severe clinical symptoms) according to an established evaluation scheme of the University Clinic for Swine of the University of Veterinary Medicine, Vienna [37]. While the body temperature of all animals was also monitored daily, the body weight was assessed at the time point of arrival and on study days 0–7, 9, 14, 21 and 28. Blood and fecal samples as well as nasal and oral swabs were taken on the study days 0, 3, 5, 7, 14, 21 and 28/29. Urine samples (spontaneous urine samples or collected via cystocentesis) were obtained on study day 3 from most animals. All animals were euthanized with T61 (5.0 mg/mL tetracaine hydrochloride, 50 mg/mL mebezonium iodide and 200 mg/mL embutramide; 1 mL/10 kg) on study day 28 or 29 under general anesthesia (1.3 mg/kg azaperone and 10 mg/kg ketamine hydrochloride). During necropsy, organ samples were taken for molecular and pathohistological analysis. In particular, the LINDA RNA loads were analyzed in samples taken from the kidney, bladder, cerebellum, cerebrum, spinal cord, dorsal root ganglia, thymus, spleen, tonsils, lymph nodes (Lnn. inguinales, mesenteriales and mandibularis), parotid and sublingual glands, heart, lung, liver and intestinal segments from all animals.

### 2.8. Pathological Examinations

Tissue samples of the brain and spinal cord, dorsal root ganglia, liver, spleen, kidney, urinary bladder, thymus, spleen, tonsils, inguinal lymph node and mandibular gland were taken from all animals for histological examination. Additional samples of the coeliac ganglion, sciatic nerve, mesenteric and mandibular lymph nodes and parotid gland were included from the experimentally infected animals and sentinels. Five coronary sections of the brain were taken at the levels of telencephalon, diencephalon, cerebellum, mesencephalon and metencephalon. Transversal sections of the spinal cord were taken from cervical, thoracic and lumbar regions. The organ samples were fixed in 10% neutral buffered formalin, embedded in paraffin wax, sectioned at 2 µm and stained with hematoxylin and eosin (HE).

## 3. Results

### 3.1. Pathogenicity and Virulence of LINDA in Weaned Piglets

**Figure 1:**
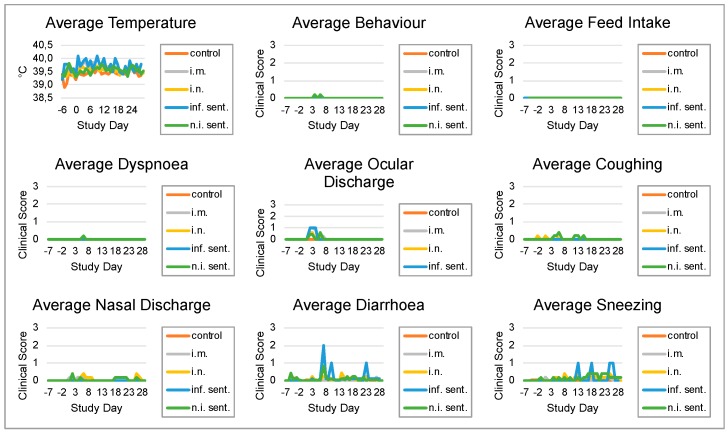
Pathogenicity of lateral-shaking inducing neuro-degenerative agent (LINDA) in immunocompetent piglets. A total of 21 piglets were divided in five groups: five negative control, five intramuscularly (i.m.) infected and five intranasally (i.n.) infected animals as well as three sentinel animals for the i.m. and three sentinel animals for the i.n. group. The animals from the i.m. and i.n. infection groups were inoculated with LINDA (1 × 10^7^ TCID_50_/mL) on study day 0. Body temperature, behavior, feed intake, dyspnea, ocular discharge, coughing, nasal discharge, diarrhea and sneezing were assessed daily and symptoms were classified by a scoring system with scores from 0 (physiological) to 3 (severe clinical symptoms). The mean of gathered parameters was calculated for the control, i.m. infected, i.n. infected, infected sentinel (inf. sent.) and non-infected sentinel (n.i. sent.) animals. No severe LINDA virus associated clinical signs were observed comparing infected and non-infected animals. However, mild fever and other signs of disease were seen in some infected animals, such as the infected sentinel animal within the i.m. group.

**Figure 2:**
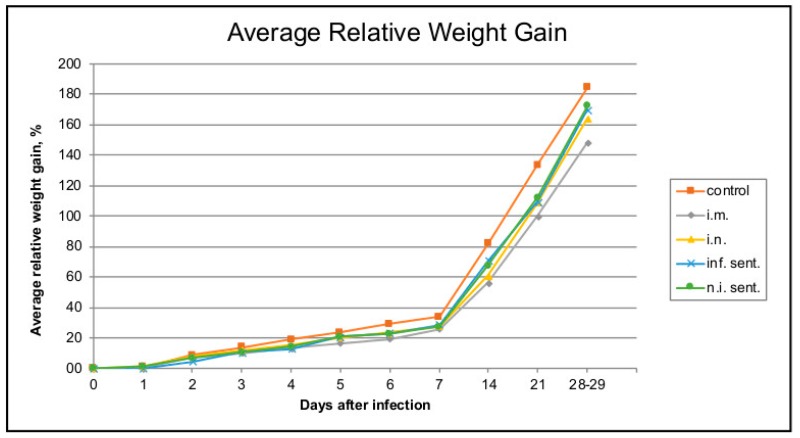
Average relative daily weight gain. Piglets were weighed on study days 0–7, 14, 21 and 28/29. The initial body weight was set as 100% value and relative weight gain was calculated for every individual at the indicated time-points. The mean value was calculated for every trial group. Infected groups show a slightly reduced weight gain compared to the negative control group after 7 days post-infection. However, the absolute differences between the groups were not significant.

The animal experiment was performed with weaned piglets to investigate the clinical effects of LINDA infection in the immunocompetent host. The animals of all three groups (infected intranasally, intramuscularly or mock), including the sentinel animals, showed normal physiological parameters and good general condition throughout the 28-day infection period. Neither the individually examined clinical values nor the additive clinical score showed major pathological changes in individual animals or significant changes between the different groups (Figure 1). Mild ocular and nasal discharge and cough were observed early after infection (study days 0 to 7) in most animals of both LINDA infected groups. However, these changes were also observed in sentinel animals of these groups that did not show seroconversion to LINDA (n.i. sent., described below). Mild fever (maximum rectal temperature of 40.7 °C) occurred in all i.m. infected animals within the first three days after inoculation. The single sentinel animal that later seroconverted showed an elevated body temperature and mild diarrhea. No differences were found between the groups in necropsy and histological examination. Gross examination revealed alveolar emphysema of the lung in animals of all the groups. Alveolar edema and pulmonary hyperemia were detected in some animals of all groups. Macroscopically, the central nervous system of all animals appeared normal. However, in the histological examination the majority of the animals from all groups (*n* = 17) showed mild, oligofocal, randomly distributed perivascular, mononuclear infiltrations and some glial nodules in the brain and/or spinal cord. A slight follicular hyperplasia of the spleen was observed in one sentinel animal of the i.n. and i.m. group. Stomach lesions such as ulcerations, hyperkeratinization or follicular hyperplasia were found in almost all animals. Mild, predominantly mononuclear, sometimes suppurating, interstitial nephritis or cortical infarction were found in many animals (*n* = 13) including animals from the non-infected control group. An interstitial lymphocytic infiltration of the mandibular gland was evident in one sentinel animal each of intranasally and intramuscularly infected group. An abscess was observed in the snout of an intranasally infected animal. The initial body weight of each animal was defined as 100% and the relative weight gain for each piglet and the average weight gain of the groups were calculated for each study day. The non-infected control group showed a slightly higher weight gain compared to the infected groups and the sentinel animals. However, the differences in weight gain between the experimental groups were neither pronounced nor statistically significant (Figure 2).

### 3.2. Replication of LINDA in the Immunocompetent Porcine Host 

**Figure 3:**
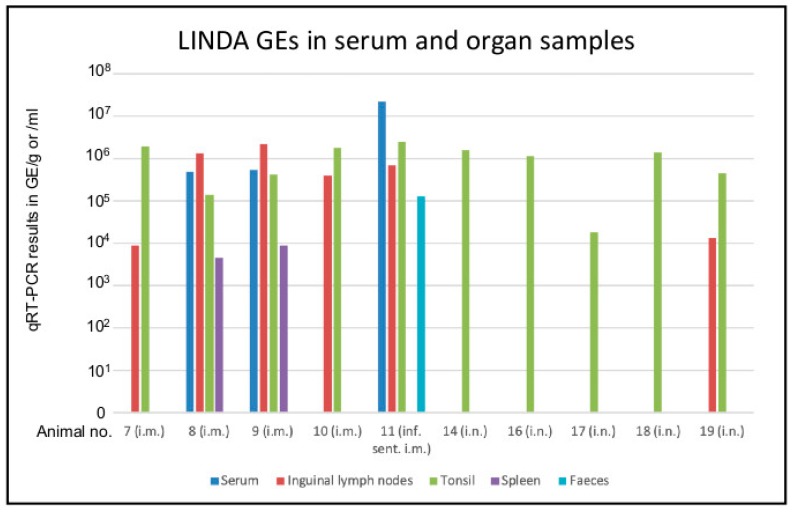
qRT-PCR results of the animal experiment. Multiple samples were analyzed for the presence of LINDA RNA before, during and after the experimental infection of piglets with LINDA. Blood and fecal samples as well as nasal and oral swabs were taken on study days 0, 3, 5, 7, 14, 21 and 28/29. Fecal samples were collected on study days 3, 7 and 14, while urine samples were only taken from most animals on day 3. Kidney, bladder, cerebellum, cerebrum, spinal cord, dorsal root ganglia, thymus, spleen, tonsils, different lymph nodes (Lnn. inguinales, mesenteriales and mandibularis), parotid and sublingual glands, heart, lung, liver and intestinal segments were sampled from all animals during necropsy on days 28/29. Most samples gave negative results for LINDA RNA. Only two i.m. infected animals showed detectable LINDA RNA loads in serum samples taken on study day 7 and one sentinel animal of the i.m. group on day 14. These serum samples also allowed the re-isolation of LINDA. Viral RNA was not detectable in urine samples and only detected in one fecal sample (animal 11, i.m. group, infected sentinel). However, the RNA of LINDA was detected in multiple samples from lymphoid organs demonstrating the presence of LINDA in the experimentally infected animals as well as in the infected sentinel animal until the end of the experiment.

Before the beginning of the experiment, blood samples of all animals were taken and tested for the presence of LINDA RNA and LINDA neutralizing antibodies. Serum samples were taken on study days 0, 3, 7, 14 and 28/29 of the experiment and analyzed in a LINDA-specific qRT-PCR assay as well as in virus isolation studies. No infectious LINDA or viral RNA were detected in the mock infected group at any time of the experiment. Most i.m. infected and all i.n. infected animals did not show a detectable viremia after LINDA inoculation. We observed a low level of viremia in two i.m. infected animals (5.0 × 10^5^ GE/mL for animal 8 i.m. and 5.5 × 10^5^ for animal 9 i.m. on study day 7) and a higher level in one sentinel animal of this group (2.3 × 10^7^ GE/mL on study day 14, Figure 3). LINDA could be isolated from each of these qRT-PCR positive serum samples. Virus shedding was assessed using oral and nasal swabs as well as fecal and urine samples. Most of these samples gave negative results in the LINDA virus-specific qRT-PCR and virus isolation experiments. The oral swabs of one of the viremic piglets from the i.m. group (study day 7) and the oral and nasal swabs from three animals of the i.n. group gave signals below assay cutoff in the qRT-PCR assay (study days 3, 7 and/or 14). The RNA loads in these swabs were very low and cell culture virus isolation was not successful. An additional conventional RT-PCR was performed on the questionable samples as described before [6]. The amplification of LINDA-specific RT-PCR products was verified by nucleotide sequencing (Figure S1). LINDA RNA was not detectable in the fecal samples obtained on study days 3, 7 and 14 from the experimentally infected animals. However, the viremic sentinel animal (number 11) from the i.m. infected group showed substantial LINDA RNA loads in the feces (1.31 × 10^5^ GE/g on study day 14) that also allowed successful virus isolation. All urine samples gave negative results in qRT-PCR and virus isolation. Multiple organs were sampled during necropsy on days 28/29 post-infection. Most organ samples gave negative results for the presence of LINDA RNA in the qRT-PCR. However, LINDA genomes were detectable in several lymphoid organs, such as the inguinal lymph nodes (*n* = 4), spleen (*n* = 2) and tonsils (*n* = 4) of animals of the i.m. infected group reaching values between 4.0 × 10^3^ and 2.3 × 10^6^ GE/g. LINDA RNA was also found in the infected sentinel animal of the i.m. group (animal 11) in the inguinal lymph nodes and spleen. Interestingly, the virus was detectable in the tonsils of all i.n. infected animals (*n* = 5), but only found in the inguinal lymph node of one of these animals (*n* = 1, Figure 3).

### 3.3. Humoral Immune Response against LINDA

**Figure 4:**
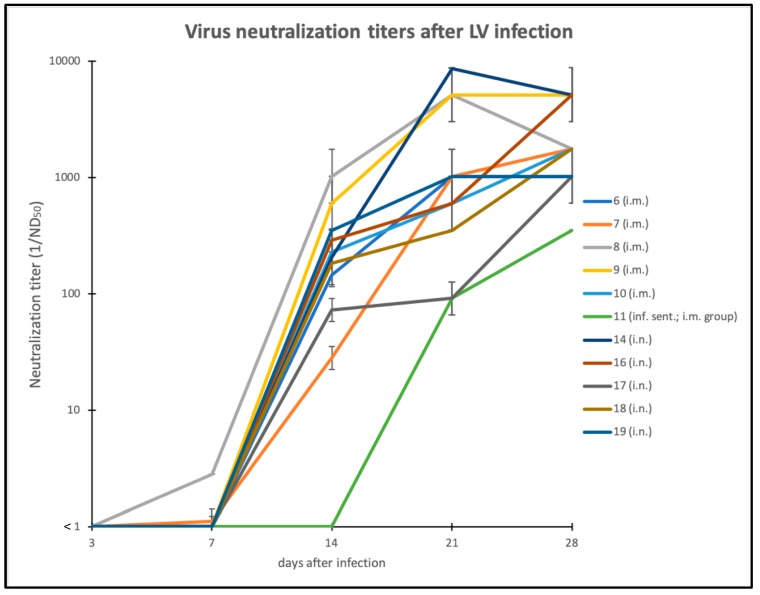
Virus neutralization activities of LINDA immune sera. The serum samples taken on study days 0, 3, 7, 14, 21 and 28/29 were tested in VNAs against LINDA. All VNAs were performed in triplicate and ND_50_ was calculated using the Spearman-Kaerber algorithm. The reciprocal ND_50_ value is presented for each serum sample including error bars for positive and negative standard deviation. All 1/ND_50_ values were less than 1 until study day 7, when the first weak neutralizing activities were measured in single animals. Significant neutralizing activities (ND_50_ > 1:10) were detected in all infected animals starting on day 14. Note the later onset of humoral immune response in the infected sentinel animal from the i.m. group on day 21.

All serum samples obtained before, during and at the end of the experiment were tested for LINDA-specific antibodies using a LINDA virus neutralization assay (Figure 4). No virus neutralizing activity was measured in the serum samples taken before the start of the experiment (ND_50_ < 1/2, below limit of detection). Sera from the mock infected group and sera from the LINDA RNA negative sentinel animals showed no virus neutralizing activity at the end of the trial. A gradual onset of humoral immune responses was observed in all infected animals (i.m. and i.n.) between days 7 and 14 post-infection, reaching peak values of up to 1/8,640 ND_50_/mL. A comparably strong reactivity of 1/1,028 was also seen in the serum sample of the i.m. infected animal 6, in which no LINDA RNA replication was detected throughout the experiment. Interestingly, the development of the humoral immune response was delayed to study day 21 in the LINDA infected sentinel animal 11. 

### 3.4. Cross Neutralization of LINDA-Immune Sera with Other Pestivirus Species

To assess antigenic relations to other pestiviruses, we characterized the LINDA-immune sera from study days 28/29 for their cross-neutralizing activities against multiple pestiviral strains. In particular, we performed VNAs with the pestivirus species BVDV-1a (strain NADL), BVDV-1b (strain NCP7), BVDV-2 (strain 890), CSFV 2.3 (strain Alfort-Tübingen), BDV-1 (strain X818), pestivirus giraffe (strain giraffe 1), BUNGO and an unpublished BVDV-3 strain. We found no neutralizing activity of the LINDA-immune sera (ND_50_ < 1/2) against any of these viruses. Additionally, we tested a porcine BUNGO convalescence serum (748-09.10-1), initially obtained for immunodetection of BUNGO infections of cultured cells. This antiserum efficiently neutralized BUNGO (BUNGO ND_50_ 1/3200) as well as LINDA in our VNAs (LINDA ND_50_ 1/1,600) but had no effect on the infection of BVDV-1, BVDV-2 and CSFV (all ND_50_ < 1/2).

## 4. Discussion

The aim of this study was to investigate the pathogenicity of LINDA during post-natal infections and to characterize the humoral immune response against LINDA in order to obtain important basic data for sero-surveillance studies. Therefore, a small-scale animal experiment was set up using LINDA infections in post-weaning piglets. In this experiment, we found no evidence of severe acute disease in weaners caused by LINDA infection. Only mild clinical signs, such as mild fever, nasal discharge and mild changes in fecal consistency, were observed in single animals without significant influence on the growth rates of these piglets. Inflammatory infiltrates and glial nodules occurred in the brain and/or spinal cord of animals from all groups including the control group. Hence, these lesions were not associated with LINDA infection and were consequently interpreted as non-specific experimental background. Other findings of necropsy could be interpreted as random findings or might represent stress-related diseases, such as the typical gastric lesions. However, productive LINDA infections were observed in all animals, which were experimentally inoculated with 1 × 10^7^ TCID_50_, regardless of the infection route used. LINDA virus excretion was detected using qRT-PCR in oronasal fluids of some infected animals and excretion via feces was documented for a single animal. Viremia was hardly detectable in the serum of most animals, while all except one experimentally infected animal (animal 6 i.m.) showed a long-lasting presence of the virus in the tonsils and/or lymphatic organs. Unfortunately, no peripheral mononuclear blood cells (PBMCs) were preserved from the experiment that could have been used for a potentially more sensitive virus detection in the blood [38]. Therefore, future studies, also including pregnant sows, will investigate whether LINDA virus is associated with PBMCs and whether the analysis of isolated PBMCs allows a more sensitive diagnosis. The infection of one sentinel animal in the i.m. group confirmed that infectious loads of virus were secreted in this simulated acute infection scenario. When we consider the positive detection of LINDA in serum, nasal secretions and feces of some infected animals, blood contacts or oronasal uptake of the pathogen may be considered as possible pathways of infection as also demonstrated by the successful artificial i.m. and i.n. infection. Since the minimum infection dose of LINDA for piglets is unknown, it is up to future studies to clarify the natural routes of infection. The clinical data are in accordance with clinical and epidemiological data about other pestiviruses, such as BVDV, BDV or CSFV, showing that mononuclear cells and lymphoid organs are the primary targets of pestivirus infections [39,40]. Most pestiviruses are well-adapted to their host species and the acute infection of immunocompetent animals usually leads to mild to subclinical disease with limited virus replication [41,42]. Severe pestiviral disease, such as abortion, malformation or neurological disorders, is mostly a consequence of intrauterine infections and responsible for the high economic losses following pestiviral infections [43]. This circumstance also explains the differences in the clinical picture between the animal experiment with immunocompetent piglets presented here and the LINDA virus outbreak in a piglet breeding farm from which LINDA virus was originally isolated [6]. Similar results were obtained in infection studies with BUNGO in weaner piglets, where few clinical signs, a short phase of viremia (3 to 10 days post-inoculation) and low levels of virus excretion were observed [17]. We conclude that LINDA is not only the closest genetic relative of Bungowannah virus but also shows similar pathogenicity in pigs. The results of our post-natal experimental infection demonstrate that the clinical picture of BUNGO and LINDA in immunocompetent animals is distinct from symptoms of piglets affected by CSFV strains of high and moderate virulence but might be similar to low virulence CSFV strains [44]. Future studies evaluating LINDA infections of the unborn fetus will be necessary to assess the potential hazard of LINDA [18,45].

The acute infection of piglets with LINDA led to the development of a strong humoral immunity starting at about seven days post-infection (Figure 4). Despite high titers of neutralizing antibodies, viral RNA persisted in the tonsils and lymphoid organs as has also been shown for other pestiviruses. This phenomenon has been described for several pestiviruses and studied in detail for BVDV pointing to a potential risk of virus transmission from convalescent animals [46]. We used the generated LINDA-antisera to evaluate antigenic cross-reactivity between LINDA and other pestiviruses. Unfortunately, we could not include APPV in these tests, because no APPV strain was available that showed the necessary infectivity in the cell culture [19]. Our VNA data clearly demonstrate that antibodies from acute LINDA infections do not provide protection against infections with classical pestiviruses. Therefore, a serological interference with established VNAs for CSFV diagnosis is unlikely. However, further studies are needed to evaluate possible false positive reactions in serological CSFV tests using a larger sample size of LINDA-antisera as well as highly reactive immune sera obtained from sows infected during pregnancy with viremic, persistently infected piglets. Our data support the hypothesis that LINDA forms an independent species (*Pestivirus L*) within the genus *Pestivirus* with a highly divergent antigenic profile. Interestingly, the high titer BUNGO-antiserum showed a strong cross-neutralization activity in VNA against LINDA, while low to absent neutralization profiles of BUNGO-antisera against other pestiviruses were observed in previous studies [47]. This result is puzzling, because it is in conflict with the results obtained with the LINDA-antisera. A possible explanation could be that the species BUNGO and LINDA form an antigenic group within the genus *Pestivirus*, sharing conserved antigenic motifs important for virus neutralization. Again, future studies may answer this question by analyzing a larger sample size of BUNGO- and LINDA-antisera in cross-protection VNAs and, even more importantly, in controlled animal experiments.

## 5. Patents

The authors B.L., L.S. and T.R. are inventors on a patent on Linda pestivirus (PCT/EP2017/084453; Isolation of a novel pestivirus causing congenital tremor).

## Supplementary Materials

The following are available online at https://www.mdpi.com/1999-4915/11/11/975/s1, Figure S1: Detection of LINDA virus by conventional RT-PCR in oro-nasal fluids.
