# Supplementary figures and images for: Clinical and Serological Evaluation of LINDA Virus Infections in Post-Weaning Piglets
Source: Viruses. 2019 Oct 23;11(11):975. doi: 10.3390/v11110975 (PMC6893756; doi:10.3390/v11110975)

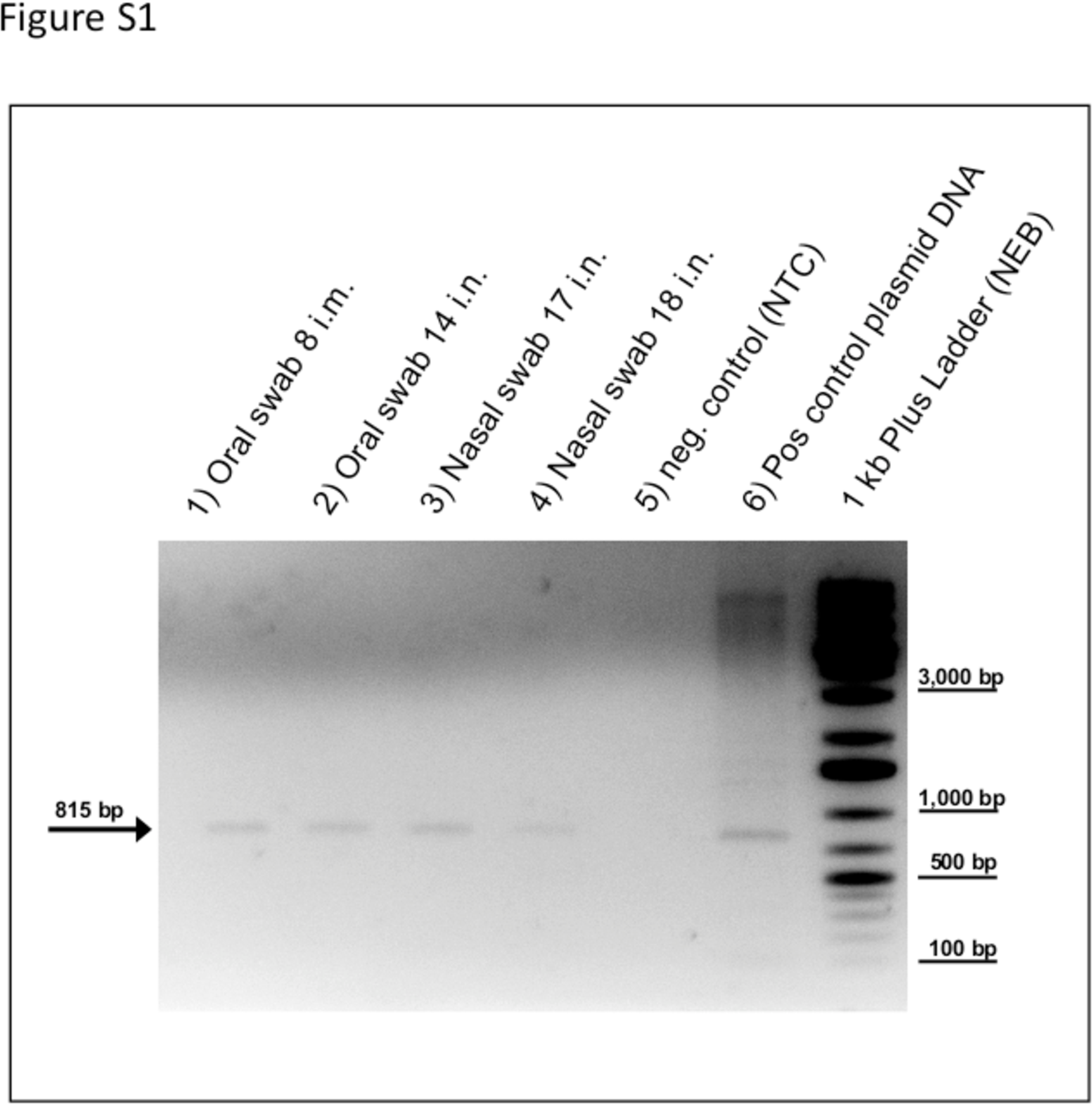

Supplement: Supplementary file 1 [file viruses-11-00975-s001.tif]
